# Burnout, Organizational Self-Efficacy and Self-Esteem among Brazilian Teachers during the COVID-19 Pandemic

**DOI:** 10.3390/ejihpe11030057

**Published:** 2021-07-23

**Authors:** Henrique Pereira, Vivianne Oliveira Gonçalves, Renata Machado de Assis

**Affiliations:** 1 Department of Psychology and Education, Faculty of Social and Human Sciences, University of Beira Interior, Pólo IV, 6200-209 Covilhã, Portugal; 2 Research Centre in Sports Sciences, Health Sciences and Human Development (CIDESD), 5001-801 Vila Real, Portugal; 3 Health Sciences and Physical Education Academic Unit, Federal University of Jataí, BR 364, KM 195, n° 3800 CEP, Jataí 75801-615, Brazil; vivianneoliveira@ufg.br (V.O.G.); renata_assis@ufg.br (R.M.d.A.)

**Keywords:** burnout, organizational self-efficacy, self-esteem, teachers, professors, Brazil

## Abstract

The purpose of this study was to assess the prevalence of, and the association between burnout, organizational self-efficacy and self-esteem, and to assess the predictive influence of burnout and organizational self-efficacy on self-esteem among Brazilian teachers during the COVID-19 pandemic. Brazilian teachers (*n* = 302) between 24 and 70 years old (*M_age_* = 46.75, *SD* = 11.02) participated in this study. Measurement instruments included a sociodemographic questionnaire, the Burnout Assessment Tool, the Organizational Self-Efficacy Scale and the Rosenberg Self-Esteem Scale. The prevalence of burnout symptoms was 3.2%, the prevalence of low occupational self-efficacy was 21.5%, and the prevalence of low self-esteem was 2.7%. Significant correlations were found between all variables under study. Hierarchical linear regression analysis showed that overall levels of burnout explained 40% of the variance of self-esteem, while together with organizational self-efficacy, it explained 55%. This study presents evidence of the impact of overall levels of burnout and self-efficacy on teachers’ self-esteem and also contributes to the body of knowledge under construction about the scenario perceived by teachers in Brazil during the COVID-19 pandemic.

## 1. Introduction

The SARS-CoV-2 virus has caused the worst global health crisis in decades, prompting the World Health Organization to declare it a pandemic in March 2020 [1]. In September 2020, Brazil was the third most affected country in the world, and while others managed to control its spread, reopen their economies, and implement precautionary measures and social distancing, Brazil was still far from reaching its epidemiological peak. The duration of the pandemic was indefinite, and its impact on the economy, and on the lives of citizens was prolonged, given that the Brazilian government promoted vertical isolation measures, instead of horizontal, which resulted in a lack of control over the movement of people, reopening of non-essential activities and an increase in COVID-19 infections.

In March 2020, the Ministry of Health of Brazil determined social isolation and the Ministry of Education suspended classes in the education system through the Decree No. 343/2020 [2], and authorized the use of information and communication technologies (ICT) to continue classes during the school year, in line with policies adopted in other nations [3]. Thus, 182,600 institutions suspended classroom classes, leaving 47 million Brazilian students in primary, secondary and higher education without classes [4].

Private schools and universities resumed classes online, based on Ordinance 2117/2019 [5], which allowed up to 40% of the theoretical educational workload on digital platforms. However, in public higher education institutions, the application of the ordinance was uneven and most resisted distance education. The public university academic year in 2020 was disrupted, and many only remotely resumed classes in December 2020.

The United Nations Educational, Scientific and Cultural Organization (UNESCO) has identified teacher confusion and stress as one of the adverse consequences of school closures, due to the abrogation of such measures, uncertainty about their duration and lack of familiarity with distance education [6,7]. This may be because uncertainties about the duration of the social isolation situation led to exhaustion and other symptoms of burnout syndrome, resulting in less confidence in their ability to do their job and manage students’ behavior [8].

Burnout syndrome is responsible for emotional overtiredness among teachers, usually because of their exhausting professional activity. Burnout, because of chronic stress experienced by professionals in the work environment, is usually related to negative feelings, which can happen with stress [9]. In fact, stress can have positive or negative facets, as the body-mind system can interpret it as a threat (distress), or as a challenge and an opportunity (eustress) [9,10].

The working conditions faced make the teaching profession one of the most vulnerable to burnout, and several factors can influence this vulnerability: exposure to stressful situations, demand for productivity, excessive workload, overcrowded classes of students, work to be done at home during overtime, little professional recognition, low salaries, limited leisure, and social moments, among others [11,12]. This generates illness, overload and compromised health, adding the personal living conditions of each teacher, with regard to financial and family commitments that need to be reconciled with work [12].

A particularly troubling result of teacher burnout is its negative influence on teacher self-efficacy [12], which is defined as a person’s belief in their abilities to achieve goals and perform tasks [11]. On the other hand, some teachers adopt a posture of resilience in dealing with the elements that can trigger burnout which, in turn, can minimize or attenuate stressors and conflicts, by using specific skills applied to problem solving, thus optimizing organizational self-efficacy [13]. This differs from person to person, according to their health conditions, personality traits and intelligence levels, ability to set goals, self-efficacy beliefs, emotional regulation and strategies used to cope with stress [14].

Some researchers have explored the relationship between burnout syndrome and professional self-efficacy, reporting that teachers who consider themselves less competent in management and discipline implementation in the classroom reported a higher level of burnout than teachers with higher levels of self-efficacy [15,16].

There is also evidence that self-esteem is related to less burnout. The perception of high self-esteem has been associated with positive characteristics such as initiative, strong coping skills, confidence, dignity, persistence in the face of challenges and a feeling of positive self-esteem [17]. According to Janssen, Schaufeli and Houkes [18], people with high self-esteem tend to be successful in life because they feel confident enough to face challenges and manage the risk of failing. Thus, it is expected that individuals with high self-esteem are less affected by burnout factors at work.

Self-esteem has been defined as an overall self-assessment, which indicates the extent to which an individual believes he/she is capable, meaningful, successful, and worthy. Psychologically, it is a state of mind that prepares a person to respond according to expectations of success, acceptance, and personal strength [17]. Different studies have shown that self-esteem is related to burnout, in a moderate way [16,18] or more strongly [19].

Worldwide, the current context demonstrates that teachers are situated in an environment favorable to mental illness due to the impacts of COVID-19 [3,6,20]. Hence, it becomes relevant to analyze and discuss the working conditions of teachers, as well as the implications of emotional aspects in the fulfillment of teaching tasks. This research, therefore, aimed to assess the prevalence of burnout levels, organizational self-efficacy, and self-esteem in a sample of Brazilian teachers, to evaluate the degree of association between these variables and to determine the predictive effect of organizational self-efficacy and burnout on the self-esteem of teachers. More specifically, the following overarching hypotheses were posed: (a) the prevalence of burnout levels among Brazilian teachers is high, and levels of organizational self-efficacy and self-esteem are low; (b) there is a strong association between these variables; and (c) organizational self-efficacy and burnout are strong predictors of self-esteem.

## 2. Materials and Methods

### 2.1. Participants

Three hundred and two Brazilian teachers between 24 and 70 years of age participated in this study (mean = 46.75; *SD* = 11.02). Overall, 55% of participants identified as female and 45% as male. The majority claimed to be married (49%) or single (27.5%), lived in urban areas (93%), and held post graduate university degrees at Ph.D. level (56.2%). Meanwhile, 55.2% said they taught at the university level, in the public sector (76.6%), and belonged to the middle socioeconomic status (70.2%). Regarding absenteeism, the vast majority of participants said that they were never or almost never absent from work in the last month (89.3%) or is the last year (72.6%). Table 1 presents the sample’s sociodemographic characteristics in further detail.

### 2.2. Measurement Instruments

The survey included four categories of questions/measures: demographic information, burnout, organizational self-efficacy and self-esteem.

Demographic information. Items included: age, gender, marital status, place of residence, educational attainment, level of teaching, sector of activity, socioeconomic status, absence from work over the last month, and absence from work over the last year.

Burnout. The Portuguese version of the Burnout Assessment Tool (BAT) was used in this study [21]. The BAT includes 22 items, measuring four core symptoms of burnout: exhaustion (eight items; e.g., “At work, I feel mentally exhausted,” α = 0.90), mental distance (four items; e.g., “I struggle to find any enthusiasm for my work,” α = 0.84), emotional impairment (five items; e.g., “At work, I feel unable to control my emotions,” α = 0.82), and cognitive impairment (five items; e.g., “At work, I have trouble staying focused,” α = 0.87). Alternatively, assuming that burnout is a syndrome, the BAT instrument enables an integrated perspective. This means that all four dimensions are interrelated and refer to the same underlying condition. All items were scored on a five-point Likert scale ranging from 1 (never) to 5 (always). Responses were averaged for each subscale. To determine overall internal reliability, a Cronbach’s α = 0.94 was obtained, demonstrating excellent internal reliability.

Organizational Self-Efficacy. The Portuguese short version of the Organizational Self-Efficacy Scale (OSES) was used [22]. It comprises 6 items, each of which is rated on a five-point Likert scale ranging from 1 (not at all true) to 5 (completely true). High values reflect high occupational self-efficacy. Items include: “I can remain calm when facing difficulties in my job because I can rely on my abilities”, “When I am confronted with a problem in my job, I can usually find several solutions”, “Whatever comes my way in my job, I can usually handle it”, “My past experiences in my job have prepared me well for my occupational future”, “I meet the goals that I set for myself in my job”, and “I feel prepared for most of the demands in my job.” To determine overall internal reliability, a Cronbach’s α = 0.90 was obtained, demonstrating excellent internal reliability.

Self-esteem. The Brazilian version of the Rosenberg self-esteem scale [23] was used. The Rosenberg Self-Esteem Scale (RSES) was developed by Rosenberg in 1965 and is an instrument used to assess global self-esteem [24]. Self-esteem is personal self-assessment, which implies a feeling of value, and encompasses a predominantly affective component, expressed in an attitude of approval/disapproval in relation to oneself. RSES consists of 10 items, with contents referring to feelings of respect and self-acceptance. Half of the items are stated positively and the other half negatively. For each statement, there are four Likert type answer options (strongly agree = 4, agree = 3, disagree = 2 and strongly disagree = 1). The sum of the responses to the 10 items provides the score of the scale whose total score ranges from 10 to 40 and obtaining a high score reflects a high self-esteem. To determine overall internal reliability, a Cronbach’s α = 0.90 was obtained, demonstrating excellent internal reliability.

### 2.3. Procedures

This research was carried out through an online website that was available between October and December 2020. Participation was voluntary, and participants were referred to a linked website created specifically for the purpose of this investigation. The first page of the questionnaire explained the objectives of the study, and informed participants about how to fill it in, how to withdraw from the study, and how to contact the authors for more information. They were also asked to read and agree to an informed consent waiver.

A total of about 1776 notifications were sent and 302 participants responded voluntarily (17% response rate). The dissemination of the survey complied with all of the ethical principles of informed consent, anonymity and confidentiality. Neither rewards nor other incentives were offered. Inclusion criteria included the following: being older than 18 years of age, being a Portuguese native speaker from Brazil, and being currently professionally active in the field of educational training. Ethical approval for this study was granted by the Ethics Committee of the University of Beira Interior (code CE- UBI-Pj-2020-088).

### 2.4. Data Analysis

Descriptive statistics were performed to describe the sample (mean, standard deviation, frequencies, and percentages). To evaluate whether there were differences between comparison groups, Student’s t-tests and one-way ANOVAs were conducted. To assess the association between the variables, Pearson correlation coefficients were calculated. Hierarchical multiple regression analyses were performed. To avoid type I errors, Bonferroni correction tests were run. All statistical procedures were conducted using the Statistical Package for Social Sciences (SPSS-version 27).

## 3. Results

Table 2 shows the results for participants’ levels of burnout, occupational self-efficacy and self-esteem. Overall, 3.2% of participants presented high levels of overall burnout, and 18.9% presented medium levels overall burnout. Meanwhile, 21.5% of participants showed low levels of overall occupational self-efficacy, and only 2.7% of participants showed low levels of self-esteem.

A correlation matrix was created using all variables to assess the levels of association among burnout, occupational self-efficacy, and self-esteem. As displayed in Table 3, significant correlations were found for all variables.

Finally, we carried out a hierarchical linear regression analysis to assess the effects of overall occupational self-efficacy and overall levels of burnout on the self-esteem of the sample. The variables “Age”, “Gender” and “Level of teaching” were added in the first block (Model I). “Overall occupational Self-Efficacy” was added in the second block (Model II). “Overall levels of Burnout” was added in the third block (Model III). The first block of analysis was not significant and only explained 3% of the variance of self-esteem, while the second block explained 41%, and the third block explained 56%, respectively, and were significant. Furthermore, as shown in Table 4, both variables were strong predictors of self-esteem.

## 4. Discussion

The main objective of this investigation was to assess the prevalence of burnout levels, organizational self-efficacy and self-esteem in a sample of Brazilian teachers during the COVID-19 pandemic. The results indicate that most of the participants have low overall levels of burnout, similar to the study carried out by Vazquez et al. [25] and Gil-Monte, Carlotto and Câmara [26]. Exhaustion, the dimension most affected in the sample of this study, represents fatigue and extreme tiredness, mentioned by some authors as the core of the burnout syndrome construct [27,28]. Other studies point out that even in samples with no burnout, the level of teacher exhaustion is high [29].

It should be noted that teaching is considered to be one of the most stressful professions, especially in problematic contexts such as those found in developing countries [26,30]. In higher education, the profession has some particularities, as teachers work in academic activities that involve, in addition to teaching, administrative and bureaucratic work, participation in congresses, research and extension projects, in addition to pressure to publish scientific papers. Consequently, the mental health and performance of these professionals has been suffering significant losses [31].

On the other hand, organizational self-efficacy plays a mediating role in burnout syndrome and in the ability to deal with stressors resulting from work overload [32]. According to Bandura [33], self-efficacy beliefs are a judgment of one’s own abilities to execute courses of action required to achieve a certain degree of performance. Hence, the results of this study indicate that the majority of the participants had moderate general levels of occupational self-efficacy; however, 21.5% manifested lower levels of self-efficacy.

Self-efficacy was negatively related to burnout, as seen in the investigation carried out by Skaalvik and Skaalvik [34]. The need to address the relationship between burnout syndrome and organizational self-efficacy was strengthened by the meta-analysis carried out by Shoji et al. [15], mainly because this association was stronger among teachers than among other occupational groups.

Consistent with other studies [30,35], there is evidence that self-esteem is related to burnout, having implications in areas such as occupational success and interpersonal relationships, in addition to a relationship between self-esteem and the symptoms of burnout syndrome. For example, Zamani Rad and Rohany [36] examined the relationship between burnout and self-esteem among future Iranian teachers and found a significant relationship between these constructs, as well as Khezerlou [37], who highlighted the importance of professional self-esteem in education and the importance of reinforcing self-esteem as part of the rehabilitation of teachers with professional exhaustion.

It was also possible to verify inverse and significant correlations between variables related to burnout syndrome (exhaustion, mental distance, emotional impairment, cognitive impairment, and general level of burnout symptoms) and general levels of occupational self-efficacy and general levels of self-esteem. In particular, variables related to burnout syndrome were negatively correlated with self-esteem, while organizational self-efficacy showed a positive correlation with self-esteem, which indicates that teachers who had higher self-esteem experienced fewer symptoms of burnout, while presenting higher levels of organizational self-efficacy.

Regarding the impact of independent variables on self-esteem, burnout explained 40% of the variance, while together with organizational self-efficacy, they explained 55%. Similar results were found by Baumeister et al. [17], Johnson et al. [38] and Zamani Rad and Rohany [36], supporting the view that teachers with high self-esteem can better deal with problems in the work environment and, consequently, reduce burnout symptoms.

Considering other studies carried out during the COVID-19 pandemic and associated problems faced by teachers directly contributing to professional exhaustion [34,39], more severe scores were expected in our study, since the percentage of teachers with high and moderate levels of burnout was low. Previous studies carried out studying burnout among Brazilian university professors [15,20], as well as among teachers from other levels of education, and according to a systematic review carried out by Montoya et al. [29], also demonstrate this trend.

In view of these results, it is necessary to consider that the highest percentage of participants in this study were university professors from the public education system, which showed resistance in the adoption of remote education, having halted face-to-face classes due to the pandemic in March 2020, and online classes resumed only in December 2020, unlike teachers in the private education system. Therefore, the fact that they were not teaching classes during the period of this investigation (October to December/2020) may explain the low level of burnout among most participants, compared to previous studies. These circumstances may also explain the fact that no significant differences were found in terms of gender, contrary to what has been reported in other studies [40,41].

### 4.1. Limitations

This study is not without limitations. First, the collection of data was completed in an online format and through self-reported measures, which can cause some bias in the results presented. Second, the cross-sectional design and non-probabilistic sampling does not allow for hypothesizing any type of causal relationship. The cross-sectional design also limits the generalization of results and does not allow observing the evolution of data as the pandemic caused by COVID-19 advances. The population object of this study is formed by professors from different levels of education, but with a higher percentage of professors from higher education, which must be taken into account in the generalization of the results.

### 4.2. Future Research

Future research could make new measurements of the variables studied when the different phases of the pandemic have passed, which would allow the comparison of variables, taking into account the evolution of the health crisis, as well as the evolution of the burnout syndrome, occupational self-efficacy and self-esteem of Brazilian teachers, focusing on the different education systems in which they operate. Thus, longitudinal studies aiming to observe changes and further develop the evaluated constructs may also be relevant in the future.

## 5. Conclusions

The results of this study indicate low levels of overall burnout compared to previous studies carried out with Brazilian teachers, given the specificities of pandemic management in the Brazilian context, as well as the resistance of public universities in relation to remote teaching. The results also reveal that the symptoms of burnout syndrome were negatively correlated with self-esteem and occupational self-efficacy, while this was positively correlated with self-esteem. Regression analysis revealed that the measured variables (general levels of burnout and general self-efficacy) were strong predictors of self-esteem.

As practical implications of this research, the contribution to the body of knowledge under construction on the perceived scenario by teachers in Brazil during the COVID-19 pandemic is highlighted. Future studies should be developed in order to monitor the evolution of burnout syndrome among Brazilian teachers and their experiences with remote learning, as well as studies related to the return to classroom teaching, as has been carried out in other countries [42], which would help educational administrators and policy makers to diagnose the sources of burnout in educational settings to prevent or reduce symptoms of burnout in teachers, fostering self-esteem and occupational self-efficacy in teachers. In addition, it can increase teachers’ awareness to develop coping strategies in order to combat burnout.

## Figures and Tables

**Table 1:** Sociodemographic characteristics (*n* = 302, *M_age_* = 46.75, *SD* = 11.02, *M_worktime_* = 18.22; *SD* = 11.28).

		*n*	%
Gender	Men	136	45
	Women	166	55
Marital Status	Single	83	27.5
	Married	148	49
	De facto union	36	12
	Divorced	35	11.5
Place of Residence	Rural	21	7
	Urban	281	93
Educational Attainment	Undergraduate degree	50	16.4
	Post graduate degree Master level	82	27.4
	Post graduate degree Ph.D. level	170	56.2
Level of teaching	Fundamental education	87	28.9
	Middle education	48	15.9
	Higher education	167	55.2
Sector of activity	Public	231	76.6
	Private	71	23.4
Socioeconomic status	Low	33	10.9
	Middle	212	70.2
	High	57	29.9
Absence from work over the last month	Never or almost never	270	89.3
	Sometimes	21	7.1
	Several times	6	2.0
	Many times, or always	5	1.5
Absence from work over the last year	Never or almost never	219	72.6
	Sometimes	67	22.3
	Several times	14	4.5
	Many times, or always	2	0.6

**Table 2:** Prevalence of burnout, occupational self-efficacy, and self-esteem.

	Low	Medium	High	M (SD)	Min-Max
Exhaustion	57.9%	34.5%	7.6%	2.80 (0.78)	1–5
Mental distance	87.3%	10.7%	2%	1.91 (0.76)	1–5
Emotional impairment	89.3%	8.7%	2%	2.38 (0.71)	1–5
Cognitive impairment	77.2%	21.8%	1%	2.40 (0.71)	1–5
Overall levels Burnout Symptoms	77.9%	18.9%	3.2%	2.45 (0.62)	1–5
Overall levels of Occupational Self-Efficacy	21.5%	57%	21.5%	3.58 (0.88)	1–5
Overall levels of Self-Esteem	2.7%	67.3%	30%	3.22 (0.57)	1–4

Note: Burnout and occupational self-efficacy scores were calculated as follows: low (1–2); medium (3); and high (4–5). Self-esteem scores were calculated as follows: low (1); medium (2–3); high (4).

**Table 3:** Correlation matrix.

	1	2	3	4	5	6	7
1-Exhaustion	-						
2-Mental distance	0.552 **	-					
3-Emotional impairment	0.643 **	0.502 **	-				
4-Cognitive impairment	0.622 **	0.502 **	0.630 **	-			
5-Overall levels Burnout Symptoms	0.905 **	0.732 **	0.825 **	0.818 **	-		
6-Overall levels of Occupational Self-Efficacy	−0.414 **	−0.465 **	−0.542 **	−0.468 **	−0.555 **	-	
7-Overall levels of Self-Esteem	−0.581 **	−0.546 **	−0.541 **	−0.565 **	−0.672 **	0.629 **	-

** *p* < 0.001.

**Table 4:** Hierarchical multiple regression analysis predicting self-esteem.

	Model I			Model II			Model III		
	*B*	*SE B*	*β*	*B*	*SE B*	*β*	*B*	*SE B*	*Β*
Age	0.008	0.004	0.160 *	−0.001	0.003	−0.013	0.000	0.003	−0.009
Gender	−0.031	0.086	−0.027	0.024	0.068	0.021	0.009	0.058	0.008
Level of teaching	0.032	0.050	0.049	0.003	0.040	0.004	0.007	0.034	0.010
Overall occupational Self-Efficacy				0.409	0.039	0.644 **	0.243	0.040	0.382 **
Overall levels of Burnout							−0.422	0.055	−0.470 **
*R2*	0.030			0.410			0.564		
*F*	1.788			29.556 **			43.659 **		

* *p* < 0.05; ** *p* < 0.001.

## Data Availability

The data presented in this study are available upon request.
